# An integrated development framework for data-driven model predictive control of thermal zones in buildings

**DOI:** 10.1016/j.mex.2025.103698

**Published:** 2025-10-30

**Authors:** Peter Klanatsky, François Veynandt, Christian Heschl

**Affiliations:** Hochschule Burgenland University of Applied Sciences, Campus Pinkafeld, Steinamangerstraße 21 7423 Pinkafeld, Austria

**Keywords:** Building simulation environment for control development, Building energy management, Model predictive control, Data-driven predictive control, Reinforcement Learning, Thermally Activated Building Structure, Smart Shading Control

## Abstract

The increasing integration of renewable energy systems into the power grid necessitates enhanced demand-side flexibility in buildings. Data-driven model predictive control (DMPC) has emerged as a promising approach for such energy management task. To advance research in this field, this paper introduces a *comprehensive simulation environment* designed to facilitate the development and evaluation of DMPC solutions for buildings. This development framework:

• encompasses a customizable zone model for multiple room configurations,

• is designed with a particular emphasis on thermally activated building structures (TABS) and solar shading control,

• integrates DMPC algorithms, with adaptable state space model structures, or using reinforcement learning algorithms, supporting various optimization architectures (centralized, decentralized…).

To ensure real-world applicability, the simulation environment has been validated using over one year of data from a living-lab office building. Validation results of the thermal zone models demonstrate good accuracy, with mean absolute errors below 0.5 °C across all zones and simulation time steps (1 and 15 min). The simulation environment exhibits robust performance in simulating complex building systems, effectively capturing the dynamics of thermally activated components and solar shading mechanisms. This versatile framework enables researchers and practitioners to address the challenges posed by increasing building complexity and growing need for decentralized control approaches.

## Graphical abstract

**Figure:**
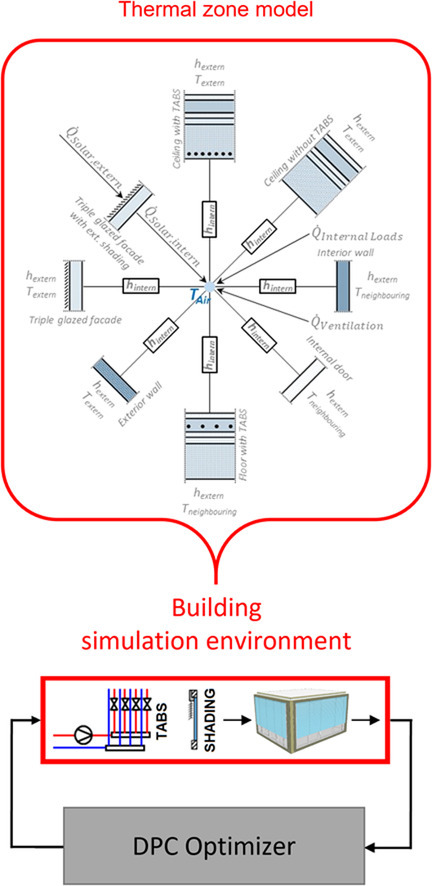

## Specifications table

**Table:** 

**Subject area**	Energy
**More specific subject area**	Method to enable the development and test of advanced control strategies for efficient building energy management.
**Name of your method**	Development framework for advanced predictive control of buildings
**Name and reference of original method**	A similar aim, of accelerating the development of advanced control strategies in buildings, is pursued by the Building optimization testing framework (BOPTEST), based on Modelica [1]. The present method proposes a specific thermal zone modelling and it is based in Matlab, to enable a direct implementation in a test building.
**Resource availability**	*-*

## Background

### 1 Challenges in building control strategies and architectures

The deployment of renewable energy increases the relevance of demand-side flexibility [2], including thermal and electrical storage systems, as well as intelligent control strategies in buildings [3]. These strategies rely on a great variety of methodologies [4,5]. Among them, Model Predictive Control (MPC) predicts the future building states to optimize control actions [6]. However, a detailed model requires high engineering and computational efforts [7,8]. Data-driven Model Predictive Control (DMPC) combines the predictive capabilities of MPC with data-driven modelling techniques [9], relying on a simplified physical model (grey-box approach) as in [10], or fully on machine learning techniques (black-box models) like in [11]. Reinforcement Learning (RL) [12] relies on algorithms such as Soft Actor-Critic (SAC) and Deep Q-Networks (DQN) [13]. However, several challenges remain, such as designing an appropriate reward function, or optimizing neural network architectures and hyperparameters [14].

As building systems grow in complexity, the structure of the optimization problem itself becomes critical [15]. Centralized optimization strategies, while theoretically capable of achieving global optima, often prove impractical due to engineering and maintenance challenges [16,17]. Decentralized and distributed optimization approaches reduce complexity but require further research to approximate global optima effectively [16,18]. Hierarchical optimization presents a compromise, with a structured decomposition of the control problem [19,20]. Agent-based solutions [21] enable autonomous decision-making at various levels of the building system [22,23].

### 2 Control system development and testing

The challenges mentioned underscore the importance of versatile simulation environments to develop and test these advanced control systems. For example, SimOpt [24] is a testbed for experimenting with simulation optimisation algorithms, across various applications [25]. In the building sector, the Building Optimization Testing Framework (BOPTEST) offers a software environment for benchmarking HVAC control algorithms [1]. BOPTEST models common building types in Modelica, enabling co-simulation to test user-developed controllers [11,26]. More recently, an open-source framework has been presented, combining devices and simulation models in co-simulation, to evaluate building control strategies [27].

Such frameworks bridge the gap between theoretical advancements and practical implementation. However, they often require interfaces and co-simulation environments which complicate seamless deployment of developed control systems in real buildings. To address this limitation, the present article proposes a development framework fully integrated in Matlab, facilitating the integration of controllers into operational buildings. This simulation environment provides access to numerical optimization methods, machine learning techniques, and RL algorithms. This supports the creation, testing and comparison of data-driven, centralized or decentralized optimization strategies for buildings.

The proposed framework uniquely incorporates Thermally Active Building Structures (TABS) and solar shading control, combining both high thermal inertia elements and rapidly changing disturbances. The authors have used this framework in a related research article [28], evaluating the performance of standard controllers and DMPCs in an office building. The present article details the underlying simulation environment, especially the building thermal zone model, with its validation using measurement data from a reference test building.

By integrating advanced control strategies with practical implementation tools, this framework contributes effectively to building energy optimisation.

## Method details

### 1 Structure and features

At its core, the framework includes a thermal zone simulation model, capable of configuring multiple room zones. The zones are modelled using a node-based approach, providing a representative model of thermal dynamics. For thermally active building components (TABS), the framework incorporates a specially developed pre-processing tool, based on Finite Element (FE) method for calculating the values of the parameters (resistances). This integration eliminates the need for external design programs, streamlining the modelling process. Users can input and configure individual zones either through a graphical user interface (GUI) or via JSON file, enhancing accessibility and ease of use. Further details on the zone model are provided in the next section 2.

The framework adopts an integral approach to account for all relevant interactions within the building. It is capable of representing the thermodynamic behaviour of individual zones, simulating the performance of heat emission systems, integrating various control systems, and performing numerical optimizations. Unlike existing building simulation tools that typically rely on interfaces for numerical optimization methods, this custom MATLAB-based tool offers greater flexibility and usability.

Furthermore, the development framework incorporates a direct API interface to integrate real-time weather forecast data and power price information. This feature, working in conjunction with a local weather station, enables a more comprehensive approach to building control and energy optimization. By combining actual weather data from the on-site station with forecast data, researchers can investigate forecast uncertainties under real-world conditions. This capability is particularly valuable for developing robust control strategies that can adapt to the inherent variability of weather patterns and energy markets.

By incorporating this real-world data –from occupant behaviour to weather patterns and energy prices–, the framework's simulations are enhanced with accurate and dynamic load profiles. This comprehensive approach ensures that the developed control strategies are not only robust but also highly applicable to real-world scenarios, capable of adapting to the complex and ever-changing conditions of actual building operations.

The development framework's flexible architecture facilitates the integration of Artificial Intelligence (AI) solutions, allowing for faster and easier configuration of simulation models. This capability, combined with its building automation features, supports automated performance-gap analysis, further bridging the gap between simulated and actual building performance.

### 2 Thermal zone model

The developed simulation model considers the one-dimensional differential equation for the heat transfer through conduction in the building components:

(1) $$\frac{\partial \rho cT}{\partial t}=\frac{\partial }{\partial x}\lambda \frac{\partial T}{\partial x}+S$$

To solve the partial differential equation the finite volume discretization with a fully implicit formulation for the discretization in time is used.

The heat transfers between the surfaces of the building components and the surroundings (ambient air, room air node) are modelled by one dimensional transfer functions, with constant overall heat transfer coefficients h (convective and radiative).

The heat losses through ventilation ($\overset{˙}{\underset{ventilation}{Q}}=\;dm_{sa}h_{sa}-dm_{ea}h_{ea}$) and the thermal inertia effects of the room air (dU), as well as further heat sources $\overset{˙}{\underset{Internal\;Loads}{Q}}$ (e.g. persons, lighting, …) were taken into account in the energy balance (Eq. (2)).

(2) dQ=dU

**Fig. 1:**
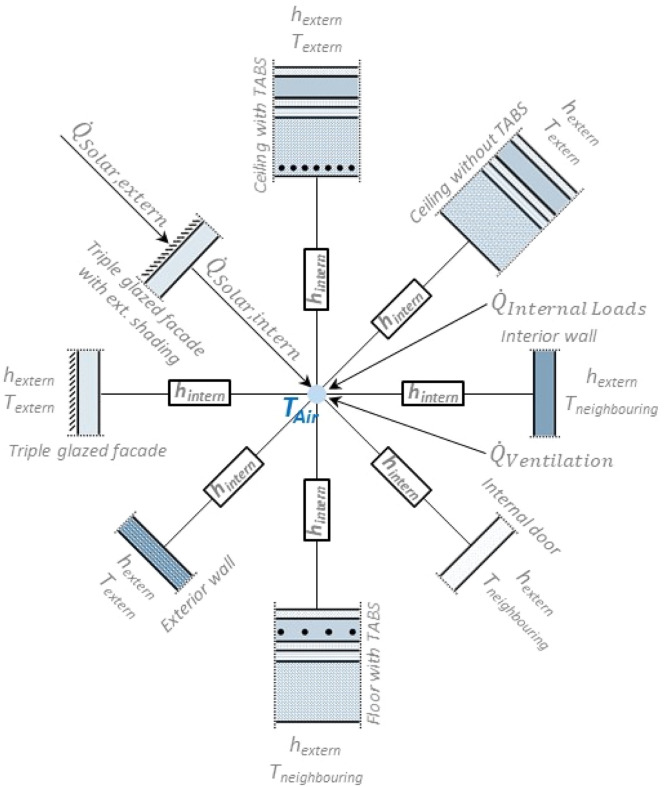
Basic scheme of the energy balance for the internal air temperature node T_Air._

Fig. 1 shows the thermal balance of the room air temperature node, including the heat transfers through the building components, as well as from the ventilation and the solar radiation.

### 3 Heating and cooling energy distribution system: TABS model

To model the TABS as static surface heating and cooling systems, all heat transfer contributions –conduction, convection, infrared radiation and solar radiation– should be considered [29]. Nevertheless, a suitable model depends on the application, as no universally valid model could be identified so far [29]. If the aim is to determine the overall performance of the system, a simple model is acceptable [30]. In the simulation environment of this study, a single heat transfer coefficient is used to model the convective and radiative heat transfer. In the case of constant supply temperatures for heating and respectively for cooling, the linearization of the radiative heat transfer is expected to be acceptable. The validation of the model of the simulation framework confirms that the hypothesis is acceptable in that case.

For the heat transfer from pipes to concrete, a shape factor approach is used, as proposed by Koschenz et al [31] and Wenig et al [32]. In this model, the general energy equation is modified and coupled to the concrete. The model from Koschenz and Lehmann is one dimensional only, which is a strong assumption. It relies on three resistances: $R_{w}$ the thermal resistance due to the convective heat transfer from the water to the inner surface of the pipe, $R_{r}$ the thermal resistance related to the conduction heat transfer from the inner side of the pipe to the outer one, and $R_{x}$ the thermal resistance connecting the temperature at the external side of the pipe with the temperature of the fictitious pipe.

### 4 Applications overview

This framework is designed to accommodate a diverse range of control approaches, enhancing its versatility and applicability. Naturally, the framework integrates standard control algorithms, such as two-point controllers and outdoor temperature-guided supply temperature controllers. This integration allows for comprehensive performance comparisons between advanced control strategies and conventional control methods under consistent conditions.

**Fig. 2:**
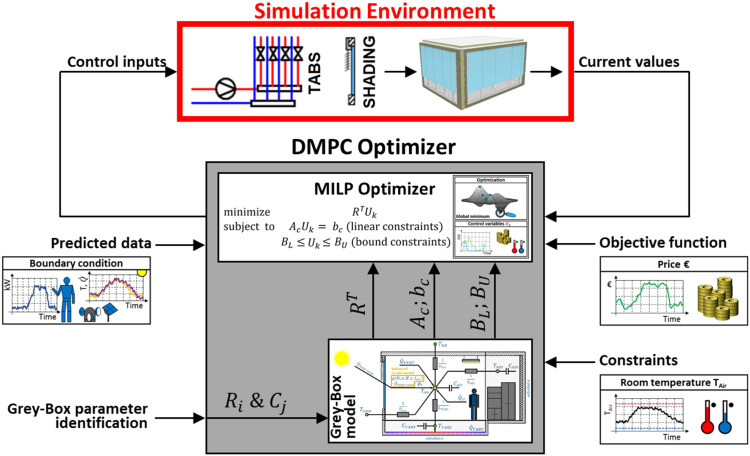
Development framework with the simulation environment and integrated DMPC-controller. This article focuses on the simulation environment (marked in red). Another article details the formulation of the DMPC optimiser [34]. The related research article shows an application of the entire development framework [33].

Most importantly, it integrates Data-driven Model Predictive Control (DMPC) algorithms as illustrated in Fig. 2. Firstly, it supports DMPC algorithms based on grey-box models with customizable resistance-capacitance model structures (R_i_C_j_) that can be individually configured to suit specific requirements. These models benefit from automatic parameter identification, allowing for efficient adaptation to different building characteristics. This functionality has been used in the related research article recently published [33]. Secondly, the framework incorporates black-box models utilizing neural networks with varying architectures, enabling the exploration of more complex, non-linear system dynamics. Additionally, the framework provides native support for reinforcement learning algorithms, further expanding the spectrum of advanced control strategies that can be investigated and implemented.

The framework provides a dedicated script file, for the development of decentralized optimization strategies. This file allows for the orchestration of DMPC controllers across multiple zones, facilitating the exploration of complex, multi-zone control scenarios.

Beyond its primary function as a development tool for control strategies, the framework can also serve as a digital twin for identifying "minimal energy demand". This capability is crucial for performance gap analysis and subsequent operational optimization, bridging the gap between simulated performance and real-world operation.

### 5 Conclusion

This research presents a comprehensive development framework for advanced control strategies in buildings, with the zone model serving as its core element. The framework's ability to accurately simulate complex building dynamics underscores its potential as a powerful tool for advanced building control strategy development. Its performance is evidenced by the validation results, showing median errors consistently below 0.2 °C (see following part on Method validation).

The zone model's flexibility and accuracy form the foundation upon which various control methods such as DMPC can be efficiently developed and tested. This capability significantly accelerates the research and implementation process, allowing for rapid iteration and refinement of control strategies. The framework's integrated approach, combining thermal modelling with control algorithm development, provides a unique platform to develop and test holistic building energy management solutions.

The framework's versatility offers a robust environment for the development and evaluation of centralized as well as decentralized optimization strategies, including agent-based systems. This feature is particularly valuable as building systems grow in complexity, necessitating more sophisticated, decentralized or distributed control approaches, including DMPC and RL.

In conclusion, this development framework represents a significant step forward in the field of building energy management. By providing a flexible, accurate, and efficient platform for the development of advanced control strategies, it paves the way for more intelligent, responsive, and energy-efficient buildings. As the built environment continues to evolve towards greater sustainability and integration with smart energy systems, tools like this framework will play a crucial role in shaping the future of building control and energy management.

## Method validation

To ensure the accuracy and real-world applicability of the framework, the “Simulation Environment” has been validated using measurement data from the living-lab environment, a real experimental building featuring various thermally active building systems. While not a direct part of the framework, the real experimental building plays a crucial role in generating realistic boundary conditions influenced by actual occupants. These boundary conditions encompass internal gains from occupant presence, power consumption patterns of electrical devices, and variations in air flow rates, as well as real weather influences, most of all solar gains.

In the following, the components that support and validate this development framework are presented in details. Section 1.1 describes the living-lab environment and its role in validation. Section 1.2 outlines the measurement and forecast data used in the framework, while Section 1.3 delves into the specifics of the building simulation model, including the node-based modelling approach for individual zones. The method and the results of the zone model validation in the living-lab environment are summarized in Section 2.

## 1 Implementation within a real building

### 1.1 Living-lab environment

#### 1.1.1 Main characteristics

**Fig. 3:**
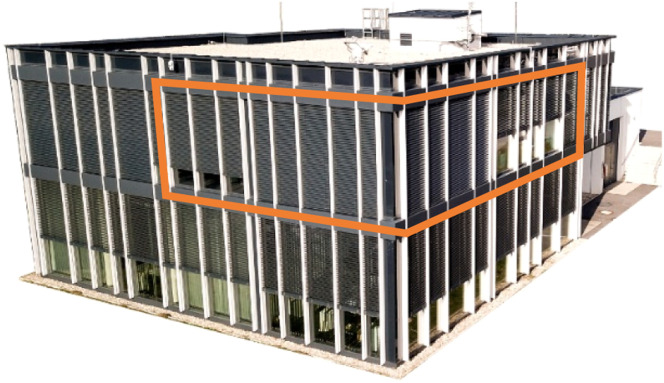
Real experimental building “ENERGETIKUM” from the point of view of South-West; orange area: chosen thermal zones.

The living-lab environment is the two-storey office building “ENERGETIKUM” with approximately 600 m² of main usable area, functioning as a living laboratory where research is conducted while the building is in everyday use. For the implementation of the zone model, three real thermal zones of the office building are considered. Fig. 3 highlights these three thermal zones, marked with an orange line.

#### 1.1.2 Location

The chosen office building is located in Pinkafeld, Austria. Key geographical characteristics of this location are listed in Table 1. The climate is temperate: Cfb type (warm temperate, fully humid, warm summer), according to the Köppen-Geiger climate classification.

**Table 1:** Geographical location of Pinkafeld.

Location: city (country)	Latitude [°]	Longitude [°]	Elevation over sea level [m]	Albedo factor [−]	Climate
Pinkafeld (Austria)	47.361 N	16.128 E	393	0.10	Cfb

#### 1.1.3 Building structure

The building envelope on the south and west sides consists of a triple-glazing façade. The east and north sides of the office building feature an insulated reinforced concrete structure. The flat roof and slabs are constructed with TABS made of massive concrete, incorporating hydraulic circuits.

#### 1.1.4 Shading system

The building automation control system integrates external venetian blinds with adjustable slats, over all glazed surface, and internal shading devices (window roller blinds), on the upper floor. Internal shading devices (vertical blinds), on the ground floor, must be operated manually.

#### 1.1.5 Technical equipment for heating, cooling and ventilation (HVAC)

For space heating and cooling, three different static heating/cooling surfaces are available. A reversible heat pump supplies the different hydraulic circuits: one in the floor, one near the ceiling surface, and one (not used in this study) deeper in the ceiling for concrete core activation. A full air conditioning system ensures hygienic air exchange rates.

#### 1.1.6 Selected thermal zones

The three selected thermal zones are named as follow:

• OFFICE 1: Thermal zone with glazed façade to the west
• OFFICE 2: Thermal zone with glazed façade to the south and west
• OFFICE 3: Thermal zone with glazed façade to the south

**Fig. 4:**
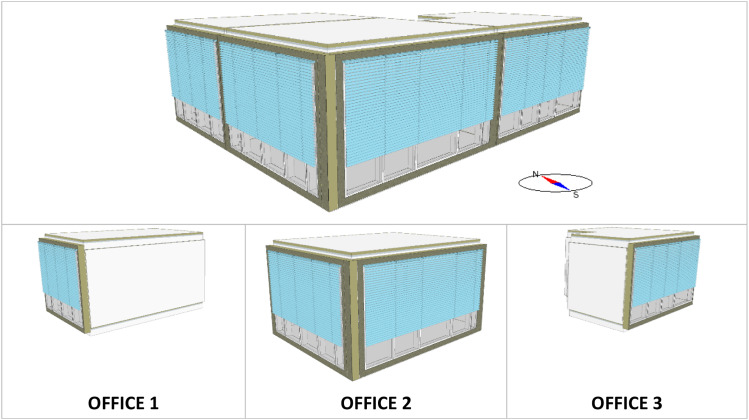
Top: 3D view of the three thermal zones on the upper floor of the ENERGETIKUM; Bottom: separate representation of each thermal zone including their names.

Fig. 4 shows a 3D view of the three thermal zones, which are usual office rooms, occupied normally during the test period.

### 1.2 Measurement data

#### 1.2.1 Monitoring system, control infrastructure

The living-lab environment is equipped with a comprehensive monitoring system that captures all relevant data, including weather conditions, room temperatures, energy demand at zone level (both electrical and thermal), occupancy counts, supply air volume, supply and exhaust air temperature and humidity, as well as supply and return temperature and mass flow for all pertinent hydraulic circuits, valve positions, setpoints, and more. Data is centrally collected through gateways, BACnet, and an edge device, ensuring a harmonised monitoring of the living-lab.

More details on the underlying infrastructure can be found in [35], which provides a similar dataset than the one used here. A precise inventory of the measurement equipment is documented in [36].

#### 1.2.2 Measurement data period

The evaluation period is a one-year dataset, from January 1st, 2019 (01.01.2019, 00:00) to December 31st, 2019 (31.12.2019, 24:00), which is available with a minutely time step. The 15-minute time step data used in this study is obtained by averaging the minutely raw variables. Data from the last days of 2018 is utilized for the start-up of the model. The start-up phase is sufficiently long to stabilize the state variables at the beginning of the evaluation period. Notably, the duration of the start-up phase is related to the building's thermal inertia. It should be checked and potentially adjusted when applying this method to other constructions.

#### 1.2.3 Overview of measurement data from the building

The required measurements are, for each thermal zone: the energy demand for heating and cooling (calculated from volume flow, supply and return temperatures), the room air temperature, the heat flow from the ventilation and the internal loads (considering power consumption and occupancy, assessed through CO_2_ measurements), as defined in [35]. The measurement data includes here the shading height and tilt slat angle, the temperature of the neighbouring rooms. The ambient temperature and horizontal global irradiance are considered as weather data. The following diagrams give an overview of key measured variables in the building.

**Fig. 5:**
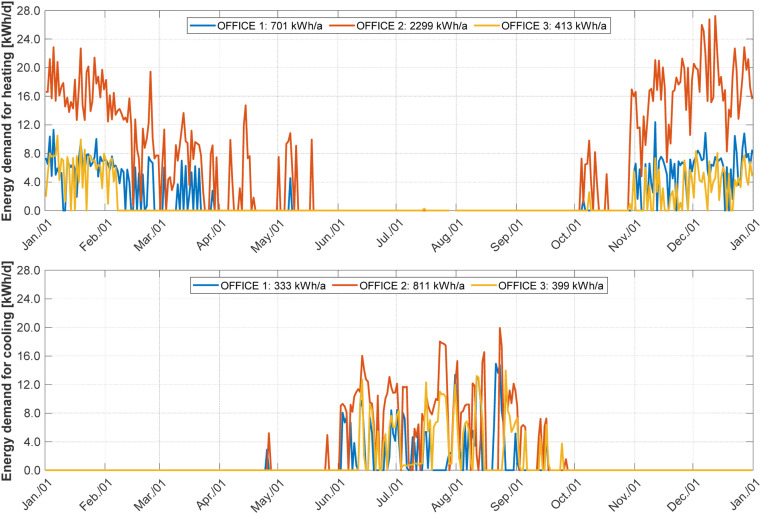
Daily energy demand for heating (top) and cooling (bottom) in each thermal zone for the year 2019.

**Fig. 6:**
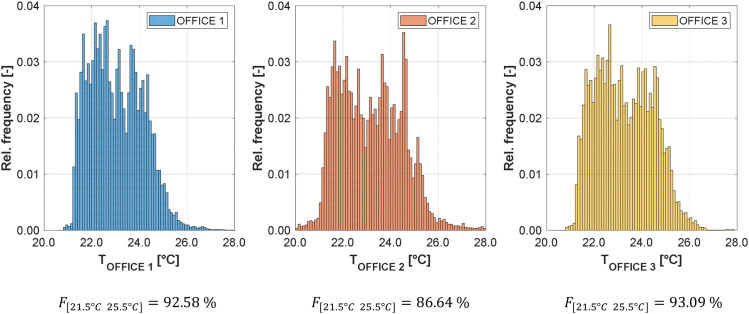
Frequency distribution histogram of the room air temperature and fraction of the time when the temperature is between 21.5 and 25.5 °C ($F_{\left[21.5^{\circ }C\;25.5^{\circ }C\right]}$).

**Fig. 7:**
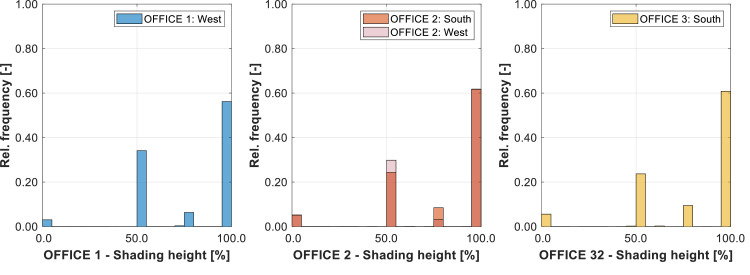
Frequency distribution histogram of the shading height. Only four positions are enabled: 0 % fully open, 50 % half-closed, 75 % three-quarter closed and 100 % fully drawn.

Fig. 5 shows the time line of the daily energy demand for heating and cooling in each zone. Fig. 6 shows the frequency distribution of the indoor air temperature, giving an indication of the thermal comfort in the rooms. Fig. 7 gives the frequency distribution of the shading height, informing on the real usage of the shading.

**Fig. 8:**
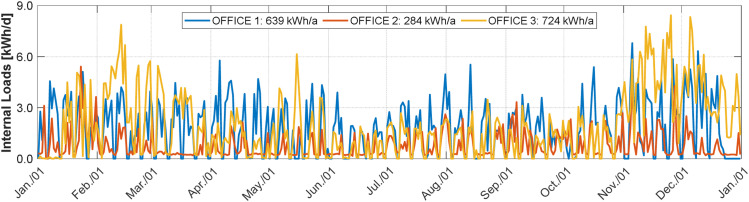
Daily energy of the internal loads in each thermal zone for the year 2019.

The daily energy of the internal loads in each zone over time is shown in Fig. 8. As can be seen, the internal loads in OFFICE 2 are lower than in the other two offices. This is partly due to a reduction in the use of artificial lighting and partly due to a reduction in the use of the office in general. The figure also shows that the internal loads in OFFICE 3 are higher between February and April and between November and December than in OFFICE 1. As a result of these higher internal loads in the year 2019, OFFICE 3 has a lower demand for heating energy in comparison to OFFICE 1 (cf. Fig. 5).

#### 1.2.4 Weather conditions

**Fig. 9:**
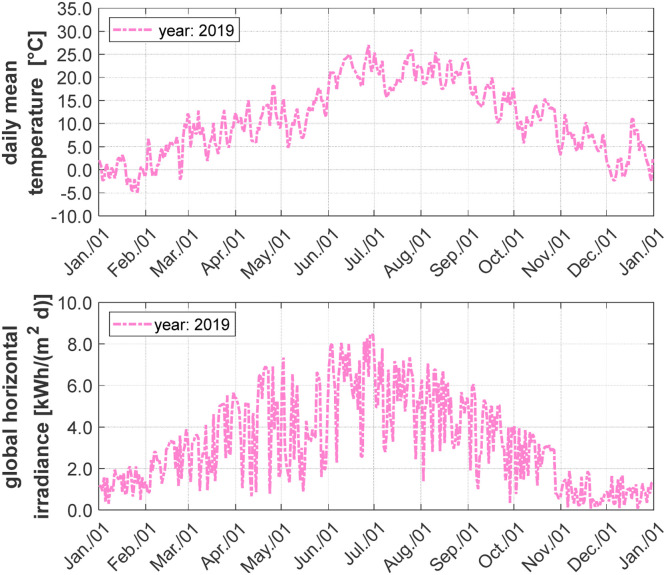
Daily average outside ambient temperature (top) and daily global horizontal irradiance (bottom) at the location of the ENERGETIKUM building in Pinkafeld (Austria) from 01/2019 to 12/2019.

The weather station installed on the flat roof of the ENERGETIKUM measures the weather conditions directly at the building location. In the following Fig. 9, the daily average of the ambient temperature and the global horizontal irradiance are shown. To give further indications on the local climatic conditions, the annual average outside ambient air temperature for 2019 is about 11.2 °C and the cumulated global horizontal solar irradiance measured over the year 2019 is $I_{global;2019}\;$= 1209 kWh/m². The calculated heating degree days (HDD) and cooling degree hours (CDH) are given in Table 2.

**Table 2:** Heating degree days (HDD) for 20 °C or 22 °C room air temperature and 12 °C outside average temperature (subscript) and cooling degree hours (CDH) for two different room air temperatures: 18.3 °C and 20.0 °C (subscript).

	01/2019 – 01/2020		01/2019 – 01/2020
HDD_20/12_	2984 K∙d	CDH_18.3_	8904 K∙h
HDD_22/12_	3388 K∙d	CDH_20.0_	6011 K∙h

#### 1.2.5 Electricity price profile

**Fig. 10:**
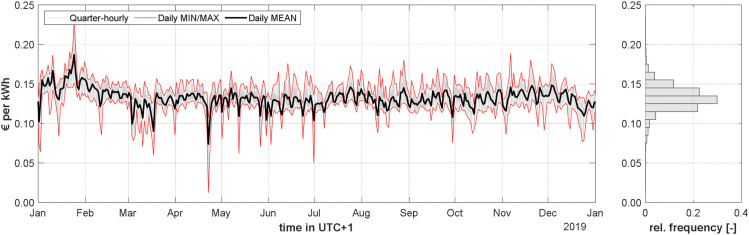
Time-dependent gross net price for the electrical energy: variable price of electricity generation and distribution, fixed price for network usage and tax in 2019.

The considered time dependent gross price of the electrical energy shown in Fig. 10 (continuous light grey line) includes the time-dependent net price of electricity, the time-independent net price for network usage (0.07 €/kWh) and the value added tax (20 %). The daily mean prices (continuous black line) and the daily minimum and maximum prices (continuous red lines) are also shown in Fig. 10. The relative frequency of the gross price of the electrical energy is shown on the right side of Fig. 10.

### 1.3 Parameters of the building simulation model

#### 1.3.1 Wall, ceiling, floor and façade construction

In Table 3, the different opaque elements of the used construction and their layers are listed.

**Table 3:** Layers of the different elements of construction – layers always from inside (room) to outside (exterior or neighbour room).

Element of construction	Short name	Layer	Material	Thickness [m]	Density [kg/m³]	Specific heat capacity [J/(kg∙K)]	Thermal conductivity [W/(m∙K)]
External wall	eW	01	Aluminium	0.002	2700	1170	200
		02	Polyurethane	0.050	35	1400	0.03
		03	Polyurethane	0.050	35	1400	0.03
		04	Aluminium	0.002	2700	1170	200
Internal wall A	iWa	01	Plasterboard	0.025	650	960	0.25
		02	Mineral wool	0.100	40	840	0.04
		03	Plasterboard	0.025	650	960	0.25
Internal wall B	iWb	01	Levelling coat	0.005	1800	1130	1.40
		02	Reinforced concrete	0.200	2400	880	2.30
		03	Levelling coat	0.005	1800	1130	1.40
Internal floor	iF	01	Floor covering	0.010	200	800	0.15
		02	Heating screed	0.080	2000	1300	1.30
		03	Insulation	0.030	100	1100	0.04
		04	Polystyrene concrete	0.120	200	1000	0.08
		05	Reinforced concrete	0.300	2400	880	2.30
External ceiling	eC	01	Reinforced concrete	0.300	2400	880	2.30
		02	Sloped concrete	0.100	2400	880	1.30
		03	EPS	0.260	150	1400	0.04
		04	Gravel	0.060	1800	1000	0.70

Another construction element is the triple-glazing façade. Each pane of the triple-glazing façade is of float glass annealed (manufacturer AGC Interpane), separated by a 14 mm wide gap filled with 90 % argon. With a low emittance coating on the inner side of the first and third pane the glazing achieves a g-value of 0.5, a U-value of 0.60 W/(m²∙K) and a visible transmittance of 70 %. The overall U-value of the triple-glazing façade is about 1.10 W/(m²∙K), which considers also the frame properties and the thermal bridges.

Regarding the internal door of dimensions 0.90 m width and 2.00 m height, a U-value of 2.40 W/(m²∙K) is assumed.

#### 1.3.2 Shading system

To ensure the shading of the triple-glazing façade, external venetian blinds are used. The distance of the slat axis to the glass is 85 mm, the slat width is 80 mm and the spacing between the slats is 62 mm. Further information on the glass and venetian blinds properties are available in previous work [37,38]. To consider the shading system in the simulation model an enhanced façade model is used to calculate the incoming solar irradiance (after shading and glass). The description of the enhanced façade model can be found in previous work of Klanatsky et al [10].

#### 1.3.3 Thermal zones

The following tables summarize the configuration of the thermal zones with the parameters used in all simulations: Table 4 presents OFFICE 1, Table 5 OFFICE 2 and Table 6 OFFICE 3.

**Table 4:** Configuration of the thermal zone OFFICE 1, with glazed façade to the west: parameters used.

Construction type	A [m²]	$h_{intern}\;$ [W/(m²∙K)]	$h_{extern}\;$ [W/(m²∙K)]	$T_{intern}$	$T_{\mathrm{exte}\mathrm{rn}}\;\;\mathrm{or}\;\;T_{\mathrm{neig}\mathrm{hbou}\mathrm{ring}}$
Internal wall A	20.16	8.0	8.0	T_OFFICE 1_	T_OFFICE 2_
Internal wall A	11.64	8.0	8.0	T_OFFICE 1_	T_CORRIDOR_
Internal wall B	20.16	8.0	8.0	T_OFFICE 1_	T_MANAGEMENT_
Internal door	1.80	8.0	8.0	T_OFFICE 1_	T_CORRIDOR_
Internal floor - FH	26.46	10.8	10.8	T_OFFICE 1_	T_OFFICE_
External ceiling	12.46	10.8	24.0	T_OFFICE 1_	T_AMB_
External ceiling - CC	14.00	10.8	24.0	T_OFFICE 1_	T_AMB_
Glazed façade W	14.08	8.0	24.0	T_OFFICE 1_	T_AMB_

**Table 5:** Configuration of the thermal zone OFFICE 2, with glass façade to the south and west: parameters used.

Construction type	A [m²]	$h_{intern}\;$ [W/(m²∙K)]	$h_{extern}\;$ [W/(m²∙K)]	$T_{intern}$	$T_{\mathrm{exte}\mathrm{rn}}\;\;\mathrm{or}\;\;T_{\mathrm{neig}\mathrm{hbou}\mathrm{ring}}$
External wall	2.78	8.0	24.0	T_OFFICE 2_	T_AMB_
Internal wall A	20.16	8.0	8.0	T_OFFICE 2_	T_OFFICE 1_
Internal wall A	9.98	8.0	8.0	T_OFFICE 2_	T_OFFICE 3_
Internal wall A	4.38	8.0	8.0	T_OFFICE 2_	T_CORRIDOR_
Internal door	1.80	8.0	8.0	T_OFFICE 2_	T_CORRIDOR_
Internal floor- FH	31.81	10.8	10.8	T_OFFICE 2_	T_MEETING_
External ceiling	12.81	10.8	24.0	T_OFFICE 2_	T_AMB_
External ceiling - CC	19.00	10.8	24.0	T_OFFICE 2_	T_AMB_
Glazed façade S	18.56	8.0	24.0	T_OFFICE 2_	T_AMB_
Glazed façade W	14.98	8.0	24.0	T_OFFICE 2_	T_AMB_

**Table 6:** Configuration of the thermal zone OFFICE 3, with glass façade to the south: parameters used.

Construction type	A [m²]	$h_{intern}\;$ [W/(m²∙K)]	$h_{extern}\;$ [W/(m²∙K)]	$T_{intern}$	$T_{\mathrm{exte}\mathrm{rn}}\;\;\mathrm{or}\;\;T_{\mathrm{neig}\mathrm{hbou}\mathrm{ring}}$
Internal wall A	16.16	8.0	8.0	T_OFFICE 3_	T_SIM03_
Internal wall A	9.98	8.0	8.0	T_OFFICE 3_	T_OFFICE 2_
Internal wall A	5.76	8.0	8.0	T_OFFICE 3_	T_TECHNICAL_
Internal wall A	10.30	8.0	8.0	T_OFFICE 3_	T_CORRIDOR_
Internal wall B	6.72	8.0	8.0	T_OFFICE 3_	T_TECHNICAL_
Internal door	1.80	8.0	8.0	T_OFFICE 3_	T_CORRIDOR_
Internal floor- FH	25.47	10.8	10.8	T_OFFICE 3_	T_MEETING_
External ceiling	11.47	10.8	24.0	T_OFFICE 3_	T_AMB_
External ceiling - CC	14.00	10.8	24.0	T_OFFICE 3_	T_AMB_
Glazed façade S	18.40	8.0	24.0	T_OFFICE 3_	T_AMB_

#### 1.3.4 Heating and cooling energy distribution system

**Fig. 11:**
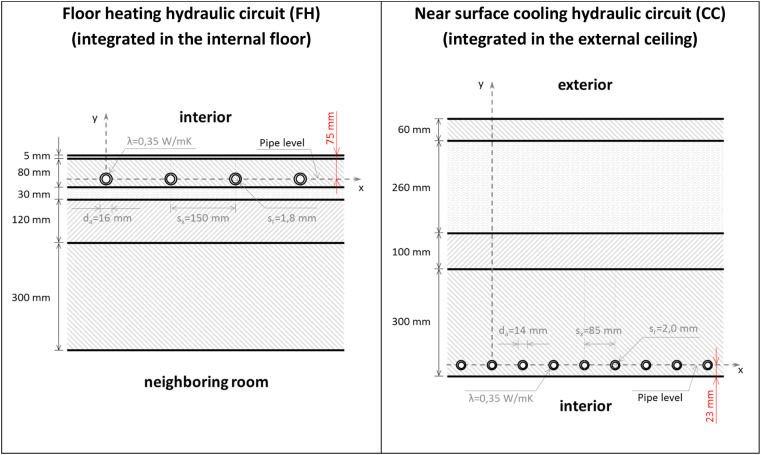
left: construction of the internal floor with the integrated pipes (under-floor heating); right: construction of the external ceiling with the integrated pipes (near-surface cooling).

The floor and ceiling constructions, shown in Fig. 11, consider the pipes with data of the hydraulic heating system (left) and the hydraulic cooling system (right).

The resistances of the surface heating and cooling systems, calculated with the mentioned pre-processing tool, are presented in Table 7.

**Table 7:** Resistances used in the model of the surface heating and cooling systems.

	Floor heating system	Near surface cooling system
	(integrated in the internal floor)	(integrated in the external ceiling)
$R_{w}$	$0.00385\;m^{2}K^{1}W^{-1}$	$0.00271\;m^{2}K^{1}W^{-1}$
$R_{r}$	$0.01739\;m^{2}K^{1}W^{-1}$	$0.01301\;m^{2}K^{1}W^{-1}$
$R_{x}$	$0.03015\;m^{2}K^{1}W^{-1}$	$0.00420\;m^{2}K^{1}W^{-1}$

#### 1.3.5 User behaviour

Since the ENERGETIKUM operates as a real office building used daily, internal gains from occupants' metabolic heat are considered. Additionally, the power consumption from equipment and lighting is taken into account.

Nevertheless, some user influences cannot be measured and are therefore not included in the developed simulation model. This includes in particular:

• door openings to the corridor (short term and/or long term)
• window openings
• precise number of occupants

## 2 Validation of the zone model with data from the living-lab environment

### 2.1 Validation method

For the validation of the developed zone model, experimental data from 2019 has been used as inputs to the simulation. This includes temperature measurements ($T_{neighbouring}$), as well as shading parameters ($h_{sh}$, $\alpha_{sh}$). The weather data ($T_{extern}$, $\overset{˙}{\underset{Solar,extern}{Q}}$), ventilation losses ($\overset{˙}{\underset{Ventilation}{Q}}$) and internal loads ($\overset{˙}{\underset{Internal\;Loads}{Q}}$) are used. For the floor heating system and the near-surface cooling system, the corrected measured heat flow ($\overset{˙}{\underset{floor}{Q}},\;\overset{˙}{\underset{ceiling}{Q}}$) data have been used.

The measured room air temperature is used as validation criteria: the deviation $\Delta T=T_{MODEL}-T_{EXP}$ between the temperature from the model $T_{MODEL}$ and the one from the experiment $T_{EXP}$ is evaluated in a statistical way.

The numerical simulations for validation have been conducted with two different time steps: 1 min (60 s) and 15 min (900 s) in each of the three thermal zones. Note that results with a 1-minute time step have 525,600 values, whereas those with a 15-minute time step have 35,040 values, both covering the full year.

### 2.2 Validation results

**Fig. 12:**
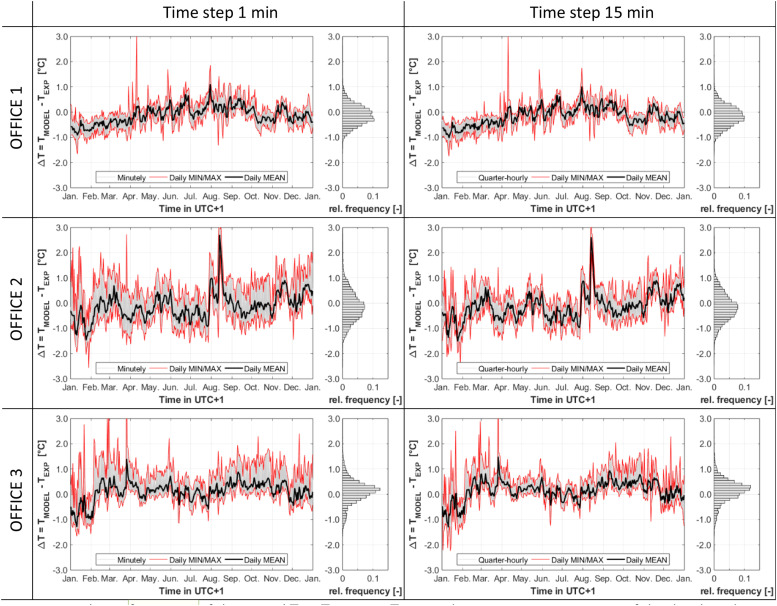
Relative frequency of the error $\Delta T=T_{MODEL}-T_{EXP}$ on the room air temperature, of the developed simulation model, for the thermal zones OFFICE 1 (with glass façade to the west), OFFICE 2 (with glass façade to the south and west) and OFFICE 3 (with glass façade to the south). Left: 1 min time step Δt=60s; Right: 15 min time step Δt=900s.

The model error for the thermal zones OFFICE 1, OFFICE 2, and OFFICE 3 is shown as time series and relative frequency histograms in Fig. 12. The statistical analysis of the model error for all three thermal zones is summarized in Table 8. The comparison of simulation results, for both time steps, shows similar outcomes. Minimal variations are observed, with better results with the 1 min time step in OFFICE 1, while the 15 min time step gives better statistical results in OFFICE 2 and OFFICE 3.

**Table 8:** Comparison of the validation results with the different time duration (Δt) steps for the three thermal zones, including: minimum min(ΔT) and maximum max(ΔT) values of the model error $\Delta T=T_{MODEL}-T_{EXP}$, several quantiles, the average $\bar{\Delta T}$, the fraction of time when the error is below 0.5 °C $F_{\left|\Delta T\right|\leq 0.5{\;}^{\circ }C}$, the mean absolute error MAE and the coefficient of determination R².

			Quantile											
ROOM	Δt	min(ΔT)	1th	10th	25th	50th	75th	90th	99th	max(ΔT)	ΔT	F_|ΔT|≤0.5_ °_C_	MAE	R²
[-]	[s]	[ °C]	[ °C]	[ °C]	[ °C]	[ °C]	[ °C]	[ °C]	[ °C]	[ °C]	[ °C]	[ %]	[ °C]	[-]
OFFICE 1	60	−1.66	−0.95	−0.61	−0.38	−0.13	0.14	0.37	0.86	3.19	−0.12	78.2	0.33	0.87
OFFICE 1	900	−1.76	−1.04	−0.67	−0.42	−0.16	0.11	0.34	0.78	3.01	−0.16	75.5	0.34	0.85
OFFICE 2	60	−2.56	−1.39	−0.87	−0.53	−0.16	0.24	0.66	1.69	5.19	−0.12	58.3	0.50	0.79
OFFICE 2	900	−2.49	−1.38	−0.83	−0.52	−0.17	0.19	0.58	1.53	4.83	−0.14	61.4	0.47	0.82
OFFICE 3	60	−1.67	−1.14	−0.44	−0.13	0.14	0.36	0.64	1.34	4.73	0.11	75.1	0.37	0.83
OFFICE 3	900	−2.24	−1.13	−0.40	−0.08	0.19	0.40	0.64	1.26	4.51	0.15	75.0	0.36	0.84

The time series reveal that all models are overall well centred and relatively precise, with slightly different behaviours in the three zones throughout time. In OFFICE 1 the model tends to underestimate the temperature in winter, while daily averages are well centred the rest of the year, with a little tendance to overestimate the temperature in summer. In OFFICE 3, the model tends to slightly overestimate the temperature throughout the day, although the daily averages are mostly centred, except in the first month of the simulation. In OFFICE 2, variations in the model performance are observed with some periods of slight underestimation –in particular the first month as in OFFICE 3–, and some periods of slight overestimation, especially on several days in August. These varying deviations may occur due to modifications in the building operation and office usage, which are not described in the model, such as open internal doors and windows or higher internal loads from occupants.

The table provides a quantified statistical analysis. For the 1-minute time step, the median (50th quantile) error is −0.125°C in OFFICE 1, −0.160°C in OFFICE 2, and 0.141°C in OFFICE 3. For the 15-minute time step, the median error is −0.161°C in OFFICE 1, −0.174°C in OFFICE 2, and 0.186°C in OFFICE 3. The median errors being all below 0.2 °C confirm that the thermal zone models are well centred. The trends observed on the time series are also confirmed, with temperatures being slightly underestimated in OFFICE 1 and OFFICE 2, while they are slightly overestimated in OFFICE 3. Looking at the minimum and maximum values, it can be seen that very few data points lie out of the time series diagrams.

With the 1 min time step, the coefficient of determination R² is between 0.79 in OFFICE 2, 0.83 in OFFICE 3 and up to 0.87 in OFFICE 1. The Mean Absolute Error (MAE) is at most 0.5 °C in OFFICE 2, 0.37 in OFFICE 3 and down to 0.33 °C in OFFICE 1. In OFFICE 1 and OFFICE 3, the larger time step of 15 min increases the MAE by 3 %, while in OFFICE 2, it raises the MAE by 6 %. The share of absolute error values below 0.5 °C ($F_{|\Delta T|\leq 0.5\;\text{°}C}$,) is at least 58 % in OFFICE 2, 75 % in OFFICE 3 and up to 78 % in OFFICE 1. These results indicate the good quality of the model, although a slightly lower accuracy is observed in OFFICE 2. This can be explained by the presence of two glazed façade orientations in this zone, instead of one.

These observations indicate that the developed zone model can effectively reproduce the air temperature in the chosen thermal zones, providing satisfactory accuracy given the measured boundary conditions and the applied enhanced façade model. It can also be concluded that simulations with the larger time step of 15 min yield good results while requiring much less computing time.

## Limitations

Looking forward, this framework still has potential for improvement and opens up several avenues for future research and development:

1. Further refinement of the zone model to incorporate additional building physics and occupant behavior models could enhance its predictive accuracy.
2. More extensive validation of the zone model in other building types and structures will help assess the applicability of the proposed model across various real applications.
3. Expansion of the framework to include a wider range of renewable energy systems and storage technologies would increase its applicability in the context of smart grids and energy communities.
4. Integration of advanced machine learning techniques, such as deep reinforcement learning, will demonstrate further adaptive and efficient control strategies.
5. Development of standardized interfaces for easy integration with building management systems would facilitate the transition from simulation to real-world implementation.
6. Exploration of multi-objective optimization techniques within the framework could address the growing need for balancing energy efficiency with occupant comfort and grid stability.

## Related research article

P. Klanatsky, F. Veynandt, C. Heschl, Data-driven model predictive control for buildings with glass façade and thermally activated building structure, Energy and Buildings 336 (2025) 115,205. https://doi.org/10.1016/j.enbuild.2024.115205.

## Ethics statements

Human persons were indirectly involved in the study as occupants of the building used for validation of the method. Their informed consent has been obtained relative to the monitoring of the living lab building and the associated research activities.

## CRediT authorship contribution statement

**Peter Klanatsky:** Methodology, Software, Validation, Investigation, Data curation, Writing – original draft, Writing – review & editing, Visualization. **François Veynandt:** Software, Writing – original draft, Writing – review & editing, Visualization. **Christian Heschl:** Conceptualization, Methodology, Validation, Resources, Writing – review & editing, Supervision, Project administration, Funding acquisition.

## Declaration of competing interest

The authors declare that they have no known competing financial interests or personal relationships that could have appeared to influence the work reported in this paper.

## Data Availability

Data will be made available on request.
